# The proteomics of lung injury in childhood: challenges and opportunities

**DOI:** 10.1186/s12014-016-9106-0

**Published:** 2016-02-29

**Authors:** Prue M. Pereira-Fantini, David G. Tingay

**Affiliations:** Neonatal Research Group, Murdoch Childrens Research Institute, Royal Children’s Hospital, Flemington Road, Parkville, VIC 3052 Australia; Department of Paediatrics, University of Melbourne, Parkville, Australia; Department of Neonatology, Royal Children’s Hospital, Parkville, Australia

**Keywords:** Proteomics, Lung injury, Paediatrics, Childhood disease, Bronchopulmonary dysplasia, Infection, Asthma, Cystic fibrosis

## Abstract

Proteomics, the large-scale study of the structure and function of proteins of a cell or organism, is a rapidly developing area of biomedical research which is perfectly suited to the study of pediatric lung injury, where a variety of samples are easily, and repeatedly, accessible including plasma (reflecting a whole body response) and broncheoalveolar lung fluid (reflecting the lungs response). When applied to pediatric lung injury, proteomics could be used to develop much needed early biomarkers of lung injury, elucidate pathological pathways and determine protein alterations associated with specific disease processes. However despite the obvious benefits and need, proteomics is rarely utilized in studies of pediatric injury. This review primarily reports on the last decade of pediatric research into proteomes associated with specific respiratory diseases including bronchopulmonary dysplasia, respiratory infection, cystic fibrosis and asthma whilst also reflecting on the challenges unique to proteomic studies of the pediatric respiratory disease population. We conclude that the number of key pathological differences between the pediatric and adult study populations inhibit inference of results from adult studies onto a pediatric population and necessitate studies of the pediatric proteome. Furthermore the disparity amongst pediatric lung disease in terms of age at onset and underlying pathological mechanism (genetic, immunological, intervention-based, developmental arrest, inhaled toxin) will require proteomic studies which are well designed, with large disease specific patient sets to ensure adequate power as well as matched controls. Regardless of causative agent, pulmonary biomarkers are needed to predict the clinical course of pediatric lung disease, status, progression and response to treatment. Identification of early biomarkers is particularly pertinent in order to understand the natural history of disease and monitor progression so prevention of ongoing lung injury and impact on childhood can targeted.

## Adult lung disease from fetal origins: the long-term impact of pediatric lung injury

Lung development can be divided into several distinct phases, starting with the embryonic phase followed by the pseudoglandular, canalicular, saccular, and alveolar phases; finally there is a prolonged phase of lung growth and maturity that is completed when the body growth stops [1]. Each of these distinct developmental phases is highly susceptible to influence by environmental stresses which may include reduced nutrient and oxygen availability, mechanical ventilator support, infection or inflammation and exposure to toxins such as tobacco smoke. By affecting lung development, early exposure to damaging environmental factors can lead to persistent alterations in lung structure and function which continue to adversely influence respiratory health throughout life. This is particularly pertinent in pediatrics, in which, unlike the adult population, respiratory illness remains the most common cause of illness in childhood. Furthermore, many serious pediatric respiratory illnesses are now not fatal in childhood and can now be considered chronic illnesses of which little is known about adult outcomes. The potential for significant impact of early lung injury on outcome in adulthood, necessitates a need for studies of pediatric lung injury mechanisms.

## Proteomics is an underutilized resource in the study of pediatric lung injury

Proteomics, the large-scale study of the structure and function of proteins of a cell or organism, is a rapidly developing area of biomedical research which is perfectly suited to the study of pediatric lung injury, where a variety of samples are available for study including tissue, broncheoalveolar lung (BAL) fluid and serum. The goal of proteomics is to provide a snapshot of all proteins in a fluid, tissue or organism [2] with proteomic techniques commonly applied to identify disease specific proteins and protein patterns in biological samples [3]. Proteomic analysis has the advantage of studying: (1) networks of proteins that provide “real-time” status of disease state, (2) modulation of protein function by diseases and drugs, (3) gene activity, (4) pathogenesis of disease and (5) the prediction of new therapies [4]. When applied to pediatric lung injury, these protein patterns could be used to develop much needed early biomarkers of lung injury, elucidate pathological pathways and determine protein alterations associated with specific disease processes. In particular, given the often rapid progression of disease in the very young, identification of early markers of disease progression, when the natural history of the disease can be altered, is essential.

Amongst the sample types available for the proteomic study of pediatric lung injury unique challenges and opportunities arise. Proteomic analysis of lung tissue provides an opportunity to directly study the proteome associated with lung injury and repair, however obtaining lung tissue in children and infants is rarely undertaken in clinical practice, technically challenging to do so as the disease site is often distal and sampling is associated with a higher risk of adverse outcomes than in adults. An alternative to tissue procurement is the study of BAL fluid, which specifically reflects the lung response. BAL is a complex mixture of soluble components such as phospholipids, neutral lipids, nucleic acids, peptides, and proteins derived from resident cells, or diffusion through the alveolar-capillary barrier [5]. The proteomic analysis of BAL samples is potentially a powerful tool for the identification of proteomes linked to specific respiratory disease states, allowing us to further our understanding of injury and repair processes within the immaturely developed lung. However, the study of BAL is not without its own challenges; obtainment of BAL whilst generally considered safe and well tolerated is technically an invasive procedure with an associated risk of bleeding, barotrauma, need for intubation and sedation, severe hypoxia, and/or bronchospasm [6, 7]. Consequently, BAL sampling is usually only feasible in children and infants receiving critical care support, limiting utility to track chronic diseases. Furthermore, many factors can affect the composition of BAL fluid and therefore may impact on the reproducibility of study results, including placement of the bronchoscope, total volume of saline instilled and risk of contamination with epithelial lining fluid [6]. These clinical and sampling concerns are circumnavigated when plasma is employed in proteomic studies. Obtainment of serum samples is considered minimally invasive and low risk making it ideal for biomarker discovery studies whilst the ability to perform repeated measurements facilitates the assessment of temporal proteome changes. However changes within serum are often very small and reflect a wide range of both peripheral and central processes and therefore pinpointing changes specific to the respiratory disease being studied can be difficult. Furthermore the complexity of serum, in which there is a huge abundance of select proteins (albumin and immunoglobulins represent 75 % of the total weight [8]) may limit the detection of lower molecular weight proteins by certain techniques such as mass spectrometry. This limitation can be partially overcome by performing fractionation prior to analysis to reduce complexity or by employing alternative discovery methods such as aptamer-based assays or SWATH-MS.

**Fig. 1:**
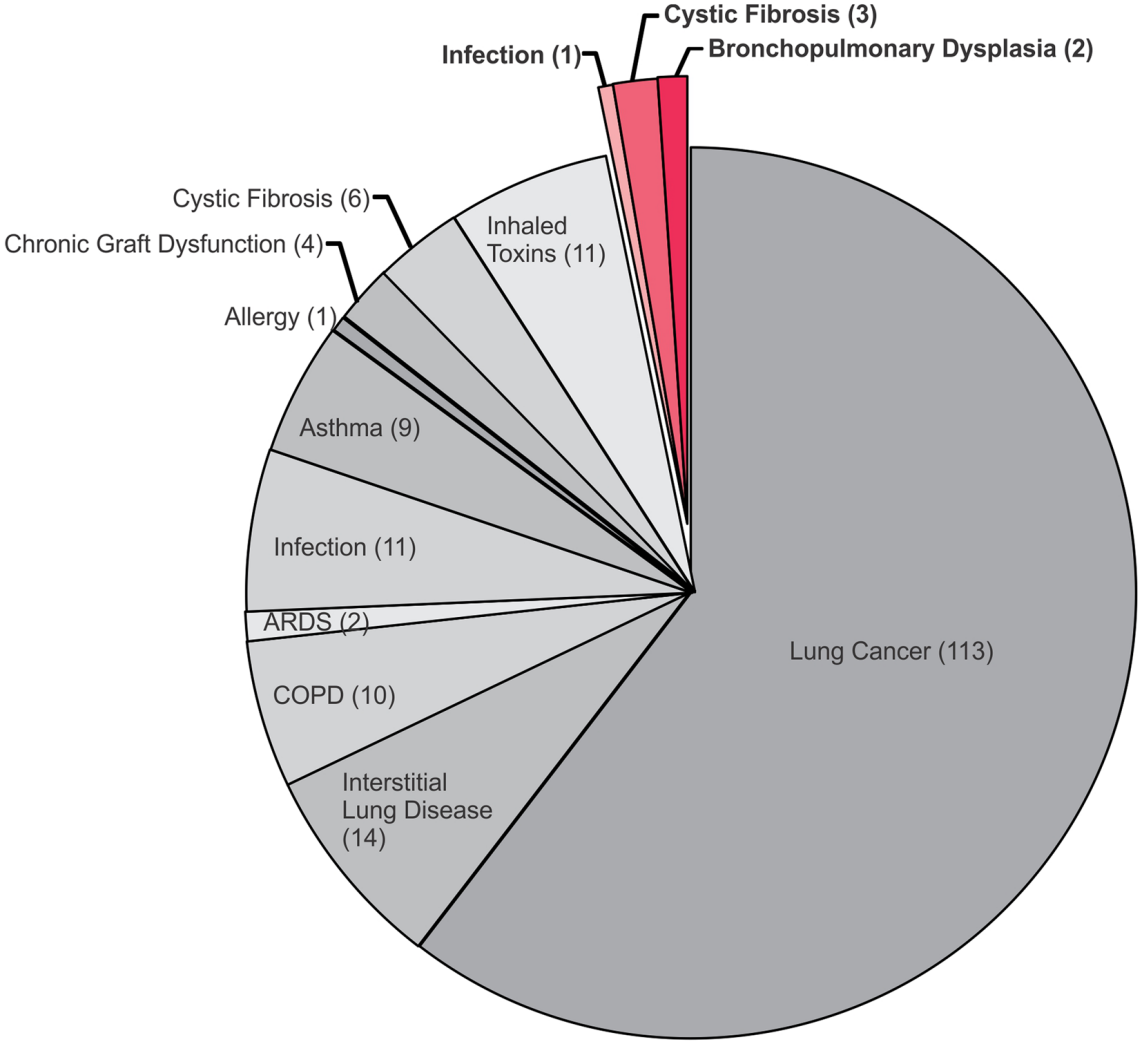
Proportion of proteomic studies of lung injury focused on pediatric lung injury (*pink*) versus adult lung injury (*grey*) over the period 2005–2015. A total of 186 proteomic studies were performed over this period

**Fig. 2:**
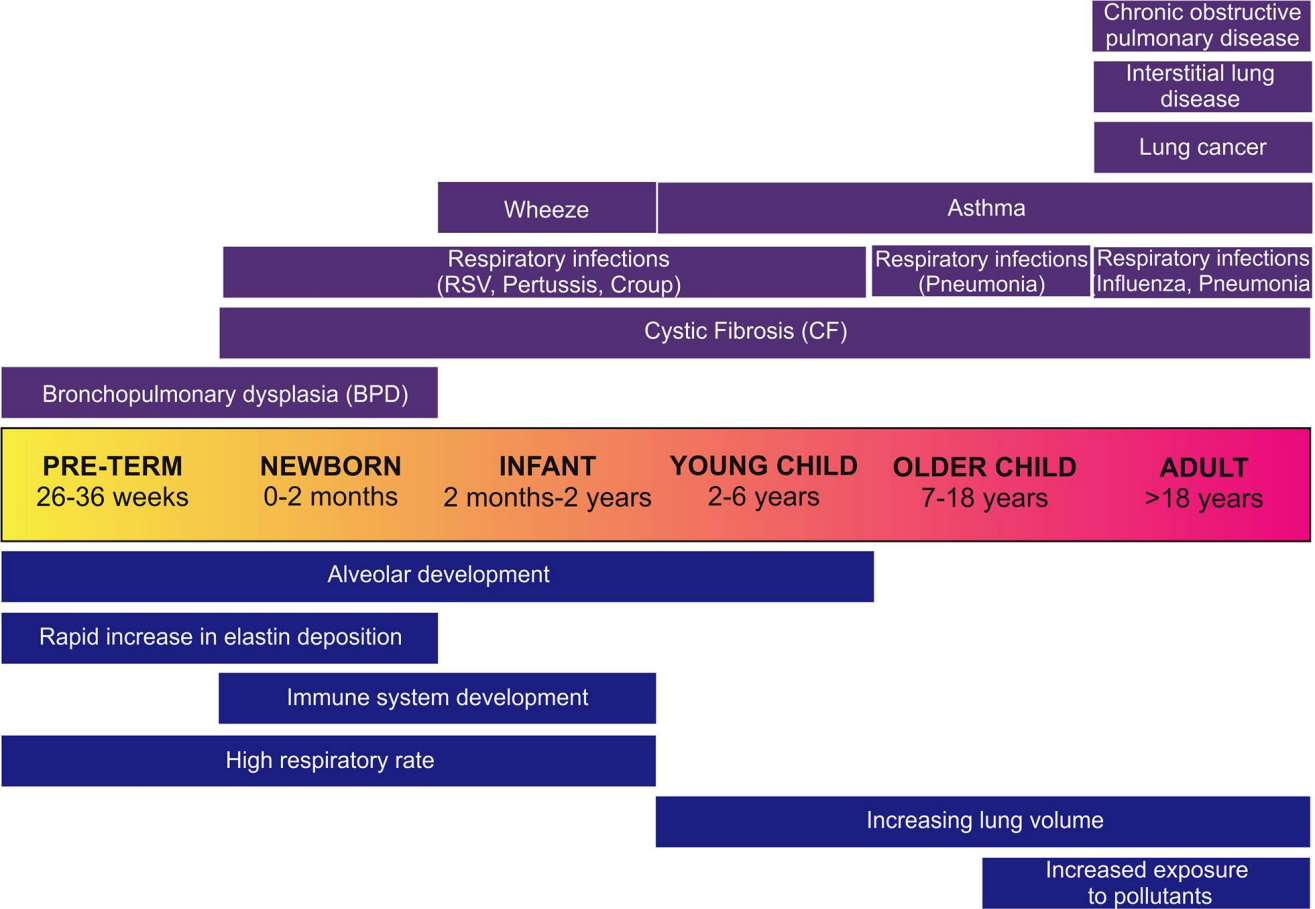
Age is a major factor in lung disease onset and likely reflects the stage of lung development

Despite the obvious benefits and need, whilst targeted ELISA-based approaches have been applied to the study of pediatric respiratory disease [9, 10], the non-targeted proteomics approach is rarely utilized in studies of pediatric lung injury. This is clearly demonstrated when reviewing the last decade of proteomic research into lung disease during which only 4 % of studies have examined childhood respiratory disorders, compared with 61 % of proteomics studies which focused on adult lung cancer (Fig. 1). The low numbers of proteomic studies focused on lung injury in childhood may reflect the unique challenges faced by researchers when studying a pediatric population. The primary of which are the complications presented when studying a maturing organ system which is undergoing profound developmental changes from pre-birth through to birth and onto adolescence (Fig. 2). Further hindering pediatric studies of lung injury is the difficulty of obtaining pediatric subjects for research, paucity of tissue samples, and the difficulty of obtaining control samples from healthy children [11, 12]. A final contributory factor may be the heterogeneity of the pediatric lung disease population which includes a variety of underlying pathologies including genetic alterations (cystic fibrosis), respiratory infection (bacterial and viral), allergy (wheeze, asthma) and intervention-induced or developmental arrest injury (bronchopulmonary dysplasia) all of which are likely to produce differing proteome profiles.

## Application of proteomic technology to the study of childhood lung disease

In a small number of studies proteomic techniques have been applied to the study of bronchopulmonary dysplasia (BPD), respiratory infection, cystic fibrosis and asthma. Details of these studies are summarized in Table 1 and included below.

**Table 1:** Overview of proteomic studies of lung injury performed in the pediatric population over the last 10 years

	Infection		Cystic fibrosis			BPD
References	Tsai et al. [24]	Neumann et al. [25]	Frischer et al. [27]	MacGregor et al. [29]	Sloane et al. [26]	Magagnotti et al. [15]
Participant age	1.9–4.8 years	2.1–15.3 years	9–28 years	5.5–7.1 years	9–13 years	25.3–31.1 days
Sample type (pretreatment)	Serum (no pretreatment)	BAL (protease inhibitor)	Bronchial tissue (extracted in sample buffer)	BAL (no pretreatment)	Sputum (DTT)	BAL (protease inhibitor)
Sample number	14 infected, 7 controls	10 infected, 7 controls	9 CF, 8 controls	39 CF, 38 controls	7 CF, 5 controls	9 BPD, 3 controls
Method employed	2-DE, MALDI-TOF	2-DE	MALDI-TOF, MALDI-TOF/TOF-MS	SELDI-TOF MS	MALDI-TOF	nLC-ESI-MS/MS
Further validation employed	Yes (ELISA, western blot)	No	No	Yes (Calgranulin A confirmed by western blot)	Yes (ELISA and western blot)	Yes (western blot)
Identified proteins	↑ haptoglobin, ↑ immunoglobulin kappa C, ↓ apolilipoprotein A-I, ↓ transthyretin	↑ α1-antitrypsin, ↓ transthyretin	↑ GRP75, mitochrondrial, ↑ Ubiquinol-cytochrome C reductase, ↑ Nidogen	↑ Calgranulin A, ↑ Calgranulin B, ↑ Calgranulin C	↑ α1-antitrypsin, ↑ myeoloperoxidase	↑ SFTP-A2, ↑ annexin-3, ↓ CLIC1, ↓ Calcyphosine

### Bronchopulmonary dysplasia (BPD)

Preterm birth is an increasing public health problem, with 15 million preterm infants born per year, of which 1 million will die [13]. Respiratory failure remains the hallmark disease of prematurity, as the predominantly canalicular or saccular, and surfactant deficient, lung is required to commence gas exchange whilst at high risk of mechanical and inflammatory injury. BPD is the chronic manifestation of lung injury in the preterm lung, and the most common form of newborn lung disease [14]. BPD is a multi-factorial disease, with pathogenesis linked to immature lung tissue, atelectasis, volutrauma and mechanical shear force injury caused by mechanical ventilation of the canalicular or saccular lung, oxidant injury, and pro-inflammatory mediators [15]. Despite improvements in neonatal care, BPD continues to occur in approximately one-third of newborns who have birth weights <1000 g and contributes to high mortality and long-term morbidity in this population [16]. Furthermore, BPD interrupts the usual alveolar developmental process well into childhood. Despite the high mortality within this population group, and a propensity for ongoing damage in survivors due to interrupted lung development [17], only a single study has examined the proteome of broncheoalveolar fluid (BAL) obtained from BPD and control infants [15]. Whilst the study numbers were low (n = 9 BPD and n = 3 controls), study participants were matched on a range of clinical parameters ensuring that any changes noted were directly related to BPD. A clear separation of the proteome was noted amongst the three gestational age groups studied, with differential expression noted between the 23–25 week group and the 26–29 week group [15]. Identified proteins included calcyphosine (*P* = 0.006), calcium and integrin binding protein-1 (*P* = 0.011), chloride channel protein 1 (*P* = 0.001), annexin-3 (*P* < 0.0001), leukocyte elastase inhibitor (SERPINB1; *P* = 0.002), and pulmonary surfactant-associated protein-A2 (STEP-A2; *P* = 0.001) [15]. To clarify if the differentially expressed proteins were linked to gestational age or to the severity of the BPD pathology, the authors employed western blotting to quantitate the protein expression of this protein panel in BAL from both severe and mildly affected BPD babies [15]. When severity of pathology was taken into account calcyphosine, calcium and integrin binding protein-1 and CLIC1, were found to differentiate between mild and severe BPD, however Annexin-3 was found to be related to development rather than extent of pathology [15].

A major contribution to BPD development is the use of mechanical ventilation and consequent development of ventilation-induced lung injury (VILI). A 2010 study reported that 62 % of extremely preterm infants (gestation <28 weeks) born in the USA received mechanical ventilation [18], and yet the underlying injury mechanisms remain largely unknown. Intriguingly, the wide-spread uptake of non-invasive modes of respiratory support for the preterm lung has not reduced BPD rates in clinical trials [19, 20] or longitudinal datasets [21]. This suggests that the mechanistic processes of BPD are more complicated than a simple mechanical interaction between a ventilator and the preterm lung. The reason why non-invasive respiratory support has not reduced BPD has not been elucidated. Mapping the VILI-associated proteome, within either serum as a reflection of the whole body response or BAL as a direct mirror of lung function, would allow mechanisms underlying VILI development to be uncovered providing important and much needed early injury biomarkers and the development of improved treatment strategies aimed at protecting the lung and minimizing damage. Importantly, proteomic analysis allows temporal measurements, accepting that the injury pathways involved in BPD are not static events but a continuum of different processes occurring over time. Unlike other organs, the lung is a mechanical organ that is undergoing continual movement (breathing) and this can’t be stopped. Therefore, any injury pathway once initiated, may not be able to be stopped or may even be exacerbated despite resolving the initial pathology (preterm birth, aeration, surfactant deficiency for example), due to the continuing movement of the lung. By employing temporal measurements, proteomics could allow the temporal mapping of injury pathways in a variety of pediatric respiratory disease situations.

### Respiratory infections

Respiratory tract infections remain the most common cause of pediatric illness, with bronchiolitis and pneumonia having major public health implications worldwide. Fortunately, at least in advanced healthcare environments, most respiratory infections are mild, self-limiting and do not require hospital admission. Despite this severe respiratory infections remain a common reason for high-dependency support in the infant population, especially in ex-premature children. Although rarely fatal, severe respiratory infection has significant associated morbidity. Alterations in the lung proteome due to severe respiratory infection have been studied in the setting of pneumococcal pneumonia infection and in the setting of respiratory infection whilst immune-compromised. Streptococcus pneumonia is the most common cause of bacterial pneumonia in children [22, 23] and the serum proteome of children with different severities of pneumococcal pneumonia (complicated or lobar) was compared with a control group [24]. Pneumococcal infection was confirmed by either culture of *Streptococcus pneumoniae* from blood or pleural effusions or a positive result of pleural pneumococcal antigen. 400 protein spots were detected across the two dimensional electrophoresis (2-DE) gels, with four protein spots differentially expressed across the experimental groups. Secondary expression validation via ELISA revealed changes were limited to upregulation of haptoglobin (*P* = 0.007) and immunoglobin kappa chain C region (*P* = 0.001) and downregulation of transthyretin (*P* = 0.007) in the complicated pneumonia group compared with the lobar and control groups [24]. All of the differentially expressed proteins are acute phase proteins and are known to take part in inflammation.

Chronic respiratory infections are a major cause of morbidity and mortality in children receiving immunosuppressive therapy for malignancies [25]. However treatment options are often limited by the inability to identify the infectious agent. Therefore Neumann et al. [25], compared the 2D-E profile of BAL samples from children with malignancies and fever not responding to broad spectrum antibiotics with a control group with the aim of classifying the varying types of lung injury and disease in these differential patients. Pathogens identified in BAL fluid included the fungi Paecilomyces and Aspergillus, the virus *Cytomegalovirus* and the bacterium *Staphylococcus epidermidis*. The study was limited to 2D-E however proteins were identified which were able to differentiate between the controls groups, malignancy with no infection and malignancy with infection. In particular, α1-antitrypsin was increased in patients with malignancies without pathogens (*P* = 0.0027) and transthyretin was decreased in the BAL of patients with pathogens (*P* = 0.0313) [25]. Ig binding factor (*P* = 0.0006) and cystatin S (*P* = 0.0030) were increased in all malignant sample sub-groups when compared with controls [25].

### Cystic fibrosis

Cystic fibrosis (CF) is the most common fatal single gene defect in Caucasian populations [3]. CF is characterized by airway inflammation, which occurs within the first months of life, chronic bacterial infection, frequent exacerbations and ultimately respiratory failure and death [3]. Although CF is diagnosed by genetic testing, CF is a multifactorial disease whose progression over time is complex and associated with various temporal events, such as infectious colonization of the lung, nutritional, environmental and even social variables. Therapy is also dependent on clinical evaluations such as lung function and radiologic changes, both of which are likely to lag behind the occurrence of established lung pathology [26]. Due to advances in the medical care of CF over the last two decades survival into middle life is now expected. Formerly purely a pediatric disease, CF is now a disease of adulthood too. Central to this has been the realization that minimizing cumulative pulmonary deterioration from the recurring cycle of infection and inflammation will ultimately help prolong the length and improve the quality of life for an individual with CF [26]. Hence proteomic studies of CF have focused on identifying protein signatures within BAL, sputum or serum which are specific to CF pathopathology with the aim of developing rapid and repeatable diagnostic and prognostic tools to assist with the clinical assessment of lung function and disease progression.

Three studies have examined alterations in the CF proteome in bronchial tissue [27], BAL or sputum. In a study of bronchial tissue obtained from patients with CF or controls, three proteins were increased in CF tissue; glucose regulated protein (GRP75; *P* = 0.002) a member of the heat shock protein family, respiratory chain enzyme ubiquinol-cytochrome c reductase complex core protein I (*P* = 0.005) which has links to hypoxia [28], and nidogen (*P* = 0.002) whose functional role is unknown. Proteomic examination of BAL fluid obtained from children with CF and controls yielded 167 proteins which were increased and 35 which were decreased in CF patients relative to controls (*P* < 0.001), however only three of the proteins were identified; s100 A8 (calgranulin A; *P* < 0.05), s100 A9 (calgranulin B), s100 A12 (calgranulin C) [29]. However the results of both of these studies may be compromised by the choice of control group, as in both studies the control group also exhibited active microbial infection.

Sloane et al. [26] further extended current CF proteome studies by comparing the protein profile of sputum from adults and children with CF. Whilst only three proteins were identified, an advantage to this study was the identification of a pediatric specific versus adult specific CF proteome. Therefore highlighting the need to study childhood samples rather than infer results from adult studies of lung disease in which the parameters are different. In particular Sloane et al., identified numerous IgG-γ_1_ heavy chain fragments within adult sputum whilst the sputum from children with CF largely only contained full length IgG heavy and light chains [26].

### Asthma

Wheezing in preschool children is very common with one in three preschool children experiencing an episode of wheezing before their third birthday [30]. In the majority of children wheezing will cease by the age of 6 years, however one-third will develop a persistent wheeze and asthma [30], and there are no known predictors. Differentiating asthma, or a prodromal state that may progress to asthma, in the preschool child is difficult. Physical examination is rarely diagnostic alone, and the gold standard diagnostic test is demonstration of altered pulmonary function and response to bronchodilators during pulmonary function testing. Although, pulmonary function testing is a valid and longstanding diagnostic tool in adults and older children they are labor and expertise intensive. In the non-compliant preschool child generating maximal expiratory flow-volume maneuvers is challenging and reliable diagnosis difficult. Hence, practically, clinicians often make the early diagnosis of asthma based on nonspecific aspects of the clinical history and examination of the child coupled with clinical acumen. Furthermore, whilst the mainstay of asthma therapy remains the use of bronchodilators and corticosteroids, not all asthma sufferers respond similarly to treatment with corticosteroids [31], and response predictors are not available. Within this context, reliable biomarkers to identify both those young wheezing children likely to develop asthma later, and target specific therapies pathways has the potential to reduce medical costs, and improve the quality of life of young wheezing children [31]. Proteomic analysis of serum or direct respiratory tract samples from preschool children with or without a wheeze may uncover new predictors of disease outcome, allow stratification of wheeze-type and allow prediction of treatment response.

## Conclusion

Proteomics, the large-scale study of the structure and function of proteins of a cell or organism, is a rapidly developing area of biomedical research which is perfectly suited to the study of the developmental and translational nature of pediatric lung injury and yet only 4 % of proteomic studies aimed at lung injury and disease have focused on pediatric lung disease.

A number of key differences between the pediatric and adult study populations inhibit inference of results from adult studies onto a pediatric population and necessitate studies of the pediatric proteome. These differences include fundamental differences in the response to disease by an immature respiratory system versus the developed lung, the concept of the pediatric lung as a dynamic changing developmental organ and disparity amongst pediatric lung disease in terms of age at onset and underlying pathological mechanism (genetic, immunological, intervention-based, developmental arrest, inhaled toxin). Such differences will require proteomic studies which are well designed, with large disease specific patient sets to ensure adequate power as well as matched controls. Regardless of the causative agent pulmonary biomarkers are needed to predict the clinical course of pediatric lung disease, status, progression and response to treatment. Identification of early biomarkers is particularly pertinent in order to alter the natural history of the disease and prevent ongoing lung injury and impact into adulthood.

## Abbreviations

BAL: broncheoalveolar fluid
BPD: bronchopulmonary dysplasia
VILI: ventilation-induced lung injury
